# Purification and Characterization of Punein, a Pomegranate PR-4 Protein Showing Structural Similarities with the Hevein Precursor

**DOI:** 10.3390/molecules30224327

**Published:** 2025-11-07

**Authors:** Lisa Tuppo, Claudia Alessandri, Laura Zaccaro, Ivana Giangrieco, Maurizio Tamburrini, Adriano Mari, Maria Antonietta Ciardiello

**Affiliations:** 1 Institute of Biosciences and BioResources (IBBR), National Research Council of Italy (CNR), 80131 Naples, Italy; lisa.tuppo@cnr.it (L.T.); ivanagiangrieco@cnr.it (I.G.); maurizio.tamburrini@cnr.it (M.T.); 2 Associated Centers for Molecular Allergology (CAAM), 00100 Rome, Italy; claudia.alessandri@caam-allergy.com (C.A.); adriano.mari@caam-allergy.com (A.M.); 3 Institute of Biostructures and Bioimaging (IBB), National Research Council of Italy (CNR), 80131 Naples, Italy; laura.zaccaro@cnr.it

**Keywords:** food allergy, Bra r 2, prohevein, Hev b 6, N-terminal amino acid sequence, primary structure, protein similarity, protein bioinformatics, barwin

## Abstract

The detection of molecules belonging to the pathogenesis-related protein-4 (PR-4) family as a cause of allergic reactions towards the pomegranate fruit has already been suggested, although information regarding their isolation and characterization is not available in the literature. The objective of this study was the purification and description of some features of a pomegranate PR-4 protein. This protein, named punein, was purified by classical biochemical methods, identified by direct protein sequencing and mass spectrometry and analyzed by bioinformatic tools. Biochemical characterization shows that punein has a molecular mass of 13.29 kDa by mass spectrometry and about 14 kDa on SDS-PAGE, and it displays a blocked N-terminus. Bioinformatic analysis highlights that its primary structure shows similarity with the allergens prohevein (containing the strong allergen Hev b 6) and Bra r 2, from latex and turnip, respectively. In particular, punein could be aligned with the C-terminal region of prohevein, which shows IgE epitope regions, the amino acid sequences of which are partially conserved in the two molecules. However, further investigations are needed to understand the clinical relevance of this PR-4 food protein and the factors affecting the concentration of specific proteins, including punein, that are recognized by the immune systems of patients sensitized to pomegranate.

## 1. Introduction

Pomegranate is a fruit increasingly consumed worldwide in raw and processed forms because of its health-promoting effects [1,2,3]. This fruit has been reported to cause allergic reactions and, more rarely, generalized anaphylaxis [4,5]. In any case, the frequency of intake and hypersensitivity towards some foods, including pomegranate, may vary in different regions [6,7]. At present, three pomegranate allergenic proteins have been registered by the World Health Organization and International Union of Immunological Societies (WHO/IUIS), namely the 9k-LTP Pun g 1 [8], the pommaclein Pun g 7 [9], and the class III chitinase Pun g 14 [10]. LTPs represent an important group of panallergens originating from plants, which are capable of causing serious symptoms, including life-threatening anaphylaxis. Pru p 3, the peach LTP, is considered to be one of the most important allergens of this family. Sensitization to Pru p 3 may cause allergic reactions to other fruits, such as cherry (Pru av 3), apple (Mal d 3), orange (Cit s 3), gold kiwi (Act c 10), green kiwi (Act d 10), and pomegranate (Pun g 1) because of cross-reactivity events [11]. In fact, cross-reactivity represents a significant risk for allergic people. It is caused by at least partial sharing of antigenic epitopes between structurally similar proteins. For instance, a high sequence identity (ranging from 86% to 100%) could be associated with the cross-reactivity between the fruit allergenic components of the gibberellin-regulated protein (GRP) family, such as peach Pru p 7, orange Cit s 7, Japanese apricot Pru m 7, cherry Pru av 7, and pomegranate Pun g 7 [12]. The allergenic pomegranate class III chitinase, Pun g 14, shows a high identity (69%) with the latex allergen hevamine Hev b 14 and raspberry Rub i chitinase (62%) [10]. Sequence similarities were also observed with other allergenic class III chitinases, such as Indian jujube Ziz m 1 [13].

In addition, potential allergenic proteins, such as the 2S albumins, were isolated from the pomegranate seed extracts and characterized [14]. The literature has also mentioned the presence of proteins in pomegranate fruit belonging to the pathogenesis-related protein-4 (PR-4) family as a cause of allergic reactions [5], although the isolation of these proteins from their natural source, to the best of our knowledge, is not yet available.

In the paper by Tuppo et al. [10], focused on the pomegranate chitinase III, some IgE binding results about a new potential allergenic protein, named Pun g 5, were shown in comparison with some pomegranate allergens. That protein was registered by us in the UniProt database as two non-adjacent fragments of ten amino acids each, contained in a protein named punein. At any rate, the terms punein and Pun g 5 identify the same protein, or isoforms of it. This protein shows some similarities with the major latex allergen prohevein (Hev b 6.01) [15], which contains an N-terminal hevein domain (Hev b 6.02) and a C-terminal barwin domain (Hev b 6.03) [16,17]. The N-terminal hevein-like domains (HLD) of class 1 chitinases from fruits such as banana, avocado, kiwifruit, and chestnut were reported to be cross-reactive with hevein and thought to be responsible for the frequent allergic reactions of latex-allergic patients to these fruits [18,19]. The studies reported in the literature about the allergenicity of the barwin domain are still scarce.

The features of pomegranate punein were not described when this IgE-binding protein was mentioned in the past [10] because its characterization was still scarce, and the description was left for a future manuscript. Therefore, the objective of this paper was the description of some properties of punein. In particular, here we describe its identification by Edman degradation chemistry and mass spectrometry experiments, a protocol for its purification from pomegranate pulp using chromatographic separations, its elution from RP-HPLC, the electrophoretic migration analyzed by SDS-PAGE under reducing conditions, and the results obtained by bioinformatic analysis of punein in comparison with homologous proteins, including prohevein.

## 2. Results and Discussion

### 2.1. Punein Detection in Pomegranate Pulp Extract (PPE)

**Figure 1:**
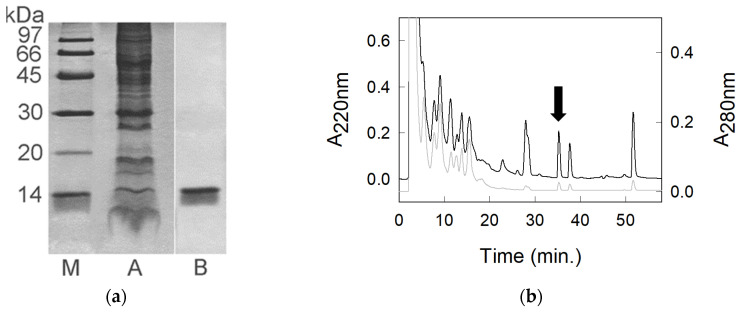
(**a**) SDS-PAGE analysis under reducing conditions. A, 20 μg of PPE; B, 5 μg of purified punein; M, molecular mass markers. (**b**) RP-HPLC analysis of about 43 μg of PPE. The absorbance at 220 nm is indicated by the black line, whereas that at 280 nm is in gray. The peak of punein is indicated by the black arrow.

To identify the proteins contained in pomegranate pulp, an aqueous extract was prepared as described in the Section 3. It showed a total protein content of about 7 mg/100 mL of pulp. Its analysis by SDS-PAGE is shown in Figure 1a (lane A). The PPE was loaded on a RP-HPLC column and eluted by a standard multistep linear gradient (Figure 1b), as described in the Section 3. For analytical purposes, the peak detected at 220 nm and eluted at 36 min was manually collected. The sample solution was concentrated and subjected to several washes with water to remove any traces of acid (trifluoracetic acid, TFA) by a rotary evaporator Savant UVS450A-230 (Thermo Fisher Scientific, Asheville, NC, USA). The contained protein was subjected to SDS-PAGE (Figure 1a, lane B) and N-terminal amino acid sequencing. The obtained result suggested that the molecule had a blocked N-terminus.

**Figure 2:**
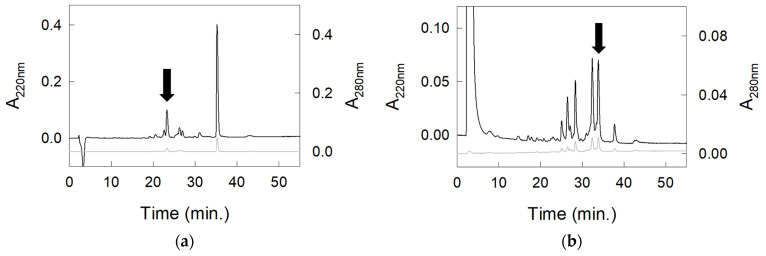
RP-HPLC separation of the digestion products of the protein eluted at 36 min from RP-HPLC. (**a**) Digestion with trypsin. (**b**) Digestion with pepsin. The absorbance at 220 nm is indicated by the black line, whereas that at 280 nm is in gray. The arrows indicate the peaks (proteolytic fragments) collected and subjected to N-terminal automated protein sequencing.

Therefore, the native protein contained in the peak eluted at 36 min and deriving from two RP-HPLC runs was subjected to fragmentation by incubation with trypsin, which produced a partial digestion. In fact, most of the protein was eluted by RP-HPLC at 36 min, which is the elution time of the undigested protein. The few tryptic peptides (Figure 2a) were manually collected. The protein fragment contained in the peak showing the highest absorbance apart from that of the undigested protein was subjected to 10 cycles of N-terminal sequencing by an automated protein sequencer. This experiment provided the sequence YHYYNPEENH. Next, the untreated protein was subjected to digestion with pepsin, which produced several fragments. These peptic peptides were separated by RP-HPLC (Figure 2b) and manually collected. One of them was loaded on the automated protein sequencer and produced the N-terminal amino acid sequence FCATWDASKP.

The two sequence fragments (YHYYNPEENH and FCATWDASKP) were used to search the UniProtKB database. However, no protein with these amino acid stretches was identified. Therefore, these two peptides were submitted to the UniProt database, which was asked to register the molecule containing them by the name of punein. UniProt registered this protein with the accession number C0HKC6.

### 2.2. Punein Purification

**Figure 3:**
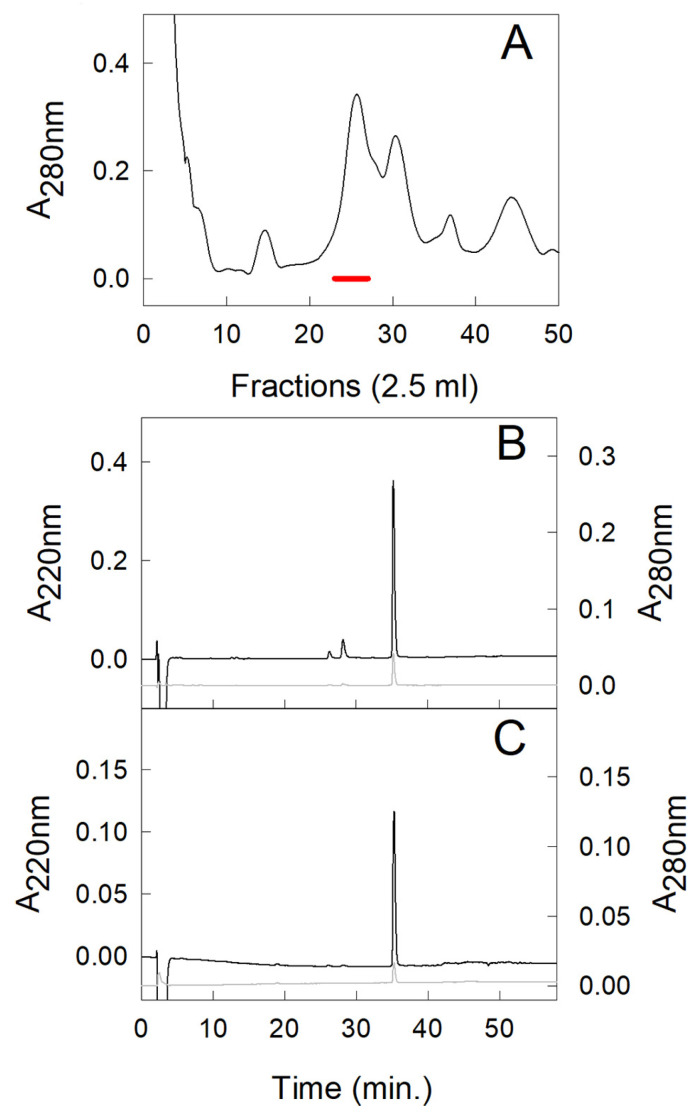
Purification of punein by chromatographic separations. (**A**) Separation by cation-exchange SP-Sepharose column of the PPE protein fraction recovered in the flow-through of the anion exchange chromatography. The red bar indicates the fractions containing punein that were pooled and used in the next step. (**B**) Separation by RP-HPLC of the pooled fractions obtained by cation exchange chromatography. (**C**) RP-HPLC of the purified punein. In (**B**,**C**) the absorbance at 220 nm is indicated by the black line, whereas that at 280 nm is in gray.

To obtain a higher amount of punein, a purification protocol was set up. In particular, 0.3 mg of punein was purified from the PPE obtained from 100 mL of the pomegranate pulp. For this purpose, the PPE was dialyzed against 10 mM Tris-HCl, pH 7.2. Next, the sample was loaded on an anion-exchange DE52 (Whatman, Brentford, UK) column, equilibrated in the same buffer. Punein was eluted in the column flow-through along with the allergenic LTP Pun g 1 [8] and pommaclein Pun g 7 [9,10], and it was monitored by RP-HPLC, registering a peak at about 36 min. This sample was then loaded on a cation-exchange SP-Sepharose column (Amersham Biosciences, Uppsala, Sweden) equilibrated in 10 mM sodium acetate, pH 5.0 (buffer A). The elution was carried out by a linear gradient from 0% to 100% of buffer B (50 mM sodium acetate, pH 5.0, containing 0.5 M NaCl) (Figure 3A). The fractions containing punein were pooled and further purified by RP-HPLC chromatography (Figure 3B). The peak eluted at 36 min of each RP-HPLC run was collected. Then, the sample solution was concentrated and subjected to several washes with water to remove any traces of acid by a rotary evaporator Savant UVS450A-230 (Thermo Fisher Scientific). The purity of the protein preparation was checked by SDS–PAGE, RP-HPLC (Figure 3C) and direct amino acid sequencing (see Section 2.3). The final protein concentration was estimated on the basis of the molar extinction coefficient at 280 nm (30,285 M^−1^ cm^−1^), calculated by the ProtParam tool, using the sequence of the predicted protein from pomegranate that, in UniProtKB, has the accession number A0A218X9Y8.

The electrophoretic separation upon SDS-PAGE of the PPE and of the purified punein are not shown, because they were very similar to those shown in Figure 1a, lane A and lane B, respectively.

### 2.3. Assessment of the Purity and Identity of the Purified Punein

The previously detected blocked N-terminus of the purified protein was confirmed by direct sequencing. Next, a new proteolytic digestion of the purified punein was performed with trypsin. The RP-HPLC separation of the digestion products was the same as that obtained before and shown in Figure 2a. Again, the tryptic fragment showing the highest absorbance was collected and sequenced for 17 cycles, which provided the sequence YHYYNPEENHWSLDDAK. The first 10 residues of this peptide of 17 amino acids had been already obtained before and corresponded to the first fragment of the protein registered in UniProt as punein. The homogeneity of the protein preparation was investigated by a collection of experimental data obtained using different methodologies. A single band and a single peak were obtained by SDS-PAGE and RP-HPLC chromatography, respectively. In addition, to exclude the presence of contaminants in the sample, as we usually do to check the purity of new proteins, an amino acid sequence analysis was carried out. Punein has a blocked N-terminus; therefore, blank cycles were recorded when four nanomoles of punein were loaded on the automated sequencer. The experiment was then repeated by loading a low amount (4 pmoles) of a control known protein (kiwellin was used in this experiment), which provided the expected sequence. This result indicated that the presence of a possible contaminant in the protein preparation could be detected, even at as low a concentration as 0.1%. However, since no contaminants were detected, the purity of the protein preparation was estimated to be about 99%. Next, the experiments of mass spectrometry (see below) detected only punein in the analyzed sample. Therefore, although PR-4 proteins belong to a multigene family [20,21], there was no indication that the protein purified by different chromatographic separations was a mix of isoforms.

**Figure 4:**
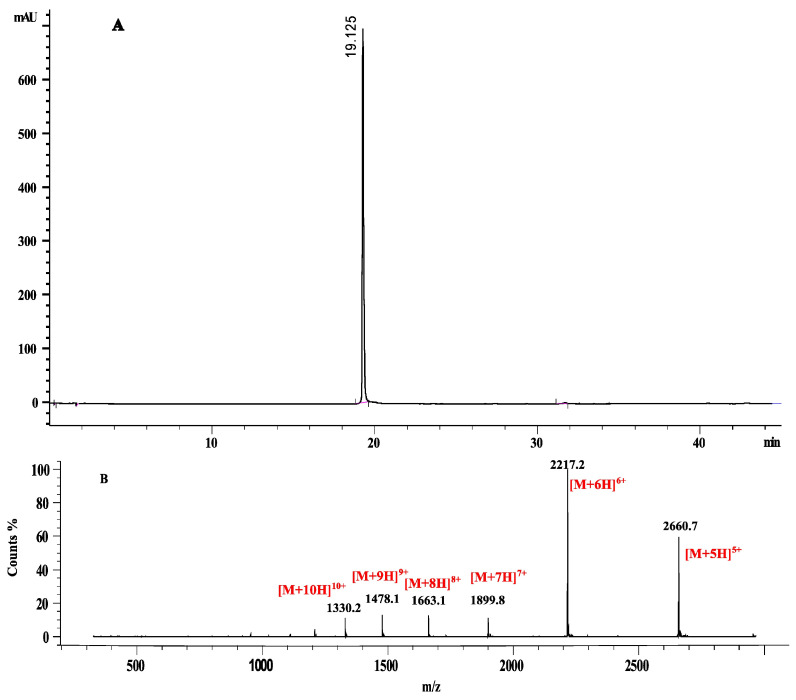
HPLC spectrum acquired at 280 nm (**A**) and mass spectrum with the charge state distribution of the protein and the individual charge states annotated (**B**).

To determine the molecular mass of the entire purified punein, mass spectrometry experiments were performed. The obtained result showed a single compound with a molecular mass of 13,298.8 Da (Figure 4).

### 2.4. Searches in the Amino Acid Sequence Databases

A similarity search was performed in the UniProtKB database (last accession was in May 2025) and was carried out using the sequence of the tryptic fragment YHYYNPEENHWSLDDAK as a query, selecting UniProtKB Swiss-Prot as the target database, 1000 as the E-Treshold, and Viridiplantae as the taxonomy; the results showed punein at the top of the list (Table 1).

**Figure 5:**
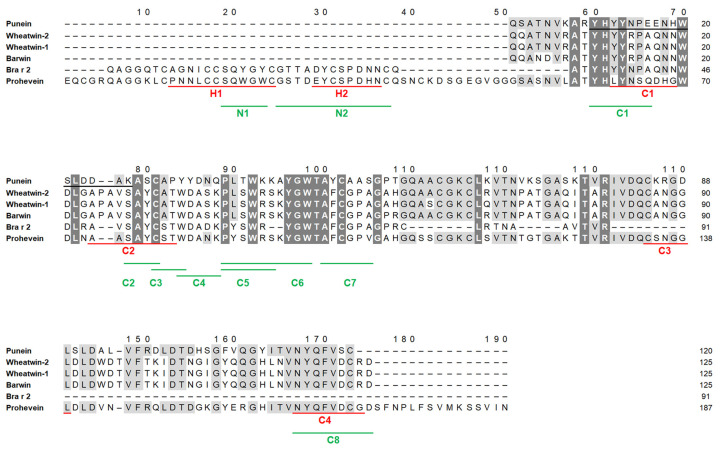
Multiple sequence alignment of mature punein (accession number A0A218X9Y8) with some PR-4 proteins. The pomegranate protein was aligned with wheatwin-2, wheatwin-1, barwin, Bra r 2, and prohevein, whose UniProt accession numbers are O64393, O64392, P28814, P81729, and P02877, respectively, using the Clustal Omega algorithm on the Expasy platform. Residues conserved in all sequences are white and highlighted in dark gray, whereas those conserved only in some sequences are black and highlighted in light gray. Residues of the purified protein obtained by direct sequencing are underlined by a black bar. Prohevein linear epitopes reported by Beezhold et al. [24] are shown by red lines and named H1–H2 and C1–C4. The epitopes described by Banerjee et al. [25] are shown with green lines and are named N1–N2 and C1–C8.

In particular, the alignment of the sequences showed that the N-terminus fragment of the registered punein had 100% identity with the first ten residues of the query peptide sequence. Conversely, the same search performed by changing the target database and using the UniProtKB reference proteomes + Swiss-Prot showed as the first match a predicted protein from *P. granatum* with the accession number A0A218X9Y8 (Table 1), belonging to the PR-4 protein family. This predicted protein sequence derives from a pomegranate genome sequence [22,23]. The alignment of the query sequence (YHYYNPEENHWSLDDAK) revealed 100% identity with the corresponding region of this predicted protein from pomegranate (Figure 5). The query sequence represented about 14% of the entire mature protein, with accession number A0A218X9Y8, also indicated as punein in Figure 5.

The molecular mass determined for punein by mass spectrometry was 13,298.8 Da. This value was in good agreement with that calculated for the predicted protein (13,297.84 kDa) starting from the residue Q, corresponding to Q51 in Figure 5, and assuming that this amino acid was a pyroglutamate. The presence of a pyroglutamate at the N-terminus makes punein similar to homologous proteins, such as wheatwin-2 and wheatwin-1, for which it was demonstrated that the N-terminal Q residue can spontaneously cyclize to pyroglutamate [26]. It is worth noting that the presence of pyroglutamate as the first residue was also in line with the experimental datum showing that the direct sequencing of the entire punein sequence does not produce results. In fact, the Edman degradation reaction cannot occur when a blocking residue is present [27].

Therefore, all together, the direct sequencing, mass spectrometry, and bioinformatics results suggested that the predicted pomegranate protein, with accession number A0A218X9Y8 [22], and punein, with accession number C0HKC6, were actually the same protein. However, the potential existence of punein isoforms with some mutations in pomegranate fruit could not be excluded since PR-4 is a multigene family of proteins, the expression of which could be regulated by a combination of different factors (biotic and abiotic stress, cell and tissue specificity, developmental regulation, plant hormone signals, innate immunity, etc.) [20,21]. The existence of isoforms could provide an explanation for the discrepancy between the amino acid sequence shown by the fragment FCATWDASKP, obtained from the peptide contained in the RP-HPLC peak eluted at 36 min (Figure 1b), then subjected to pepsin digestion, and the punein sequence in the corresponding protein region, shown in Figure 5. Therefore, it cannot be excluded that the protein sample obtained from RP-HPLC contained more than one isoform.

Then, a further experimental similarity search was carried out using the entire detected sequence of punein (accession number A0A218X9Y8) as a query, and UniProtKB Swiss-Prot selected as the database. In particular, the sequence of the mature protein, that is, the molecule without its signal peptide, which has the function of guiding the protein to its correct positioning inside or outside the cell, was used for the similarity search. Among the many resulting proteins, those classified as “allergen” were searched. This search revealed that punein also had similarities with two allergenic proteins officially registered by WHO/IUIS, prohevein from *Hevea brasiliensis* (Hev b 6) [16] and Bra r 2 from *Brassica rapa* [28] (Table 1). Both these allergens belong to the PR-4 protein family.

PR-4 proteins [21] include two main structural classes. Class 1 contains proteins with both N-terminal chitin binding domain and C-terminal barwin domain, whereas class 2 contains molecules with the barwin domain only [29]. Therefore, as also shown by Figure 5, punein, also called Pun g 5 in a previous paper [10], belongs to class 2, together with barwin, wheatwin-1, and wheatwin-2, whereas prohevein and Bra r 2 belong to class 1 of PR-4.

### 2.5. Percentage of Amino Acid Sequence Identity

Table 2 shows that, in the overlapping regions, punein shares 55%, 54%, 54%, 45%, and 55% of sequence identity with wheatwin-2, wheatwin-1, barwin, Bra r 2, and prohevein, respectively. It can be observed that the similarity values are higher when the proteins other than punein are compared to each other. The highest values are observed when wheatwin-1, wheatwin-2, and barwin are compared, probably because wheat and barley are taxonomically closer.

### 2.6. Comparison of Punein and Other PR-4 Proteins with the Corresponding Sequence Regions of Prohevein

Figure 5 shows the sequence alignment of punein with the two allergens prohevein and Bra r 2. In addition, punein is aligned with some other representative PR-4 proteins, such as the wound-induced proteins 2 (wheatwin-2) and 1 (wheatwin-1) from wheat [26,30] and the barwin protein. Barwin is a basic protein isolated from aqueous extracts of barley seeds [17]. The sequence IgE epitopes described by some authors for prohevein are shown in Figure 5, which highlights that some of them are located in regions that are at least partially conserved in punein. For instance, those described by Beezhold and collaborators [24], who reported six epitopes recognized by specific IgEs contained in the sera of 10 latex-allergic health care workers, are shown by red lines. Two epitopes were located in the N-terminal region, whereas four were located in the C-terminal one [31]. Instead, using synthetic peptides and sera from latex-sensitized health care workers, Banerjee et al. [25,32] detected ten IgE binding epitopes in prohevein. The two epitopes of the hevein domain reported by Beezhold and collaborators at least partially overlap those described by Banerjee et al. A higher number of epitopes were detected in the C-terminal region of prohevein. In fact, eight epitopes were described by Banerjee and collaborators, with some of them falling in regions similar to those reported by Beezhold and collaborators. In addition, a detailed analysis of the T-cell responses and the IgE-binding capacity of the entire prohevein, and of the separated N-terminal and C-terminal domains, was carried out by Raulf-Heimsoth and collaborators [33] using recombinant protein molecules fused with a maltose-binding protein. Interestingly, some regions of the amino acid sequence of prohevein were conserved in punein. These observations suggested that punein might have some antigenic epitopes similar to those reported for prohevein.

The similarity of punein to a region of prohevein that contains epitopes recognized by IgE is in line with previous findings reporting that this pomegranate protein can bind specific IgE antibodies from allergic patients [10]. The frequency (0.38%), calculated on the basis of the results obtained with the FABER^®^ test [34], was described in the paper by Tuppo and collaborators [10]. The FABER^®^ system allowed an in vitro test of the measurement of specific IgE in human serum or plasma. Indeed, the mentioned paper [10] was focused on the description of the pomegranate chitinase III (Pun g 14), whereas punein (there reported as Pun g 5) was only used for comparative purposes. In fact, details on punein were postponed to future publications. That paper [10] highlights that punein can bind IgE, and therefore, it is a potential allergen, the clinical relevance of which is of diagnostic interest. Nevertheless, at least in the analyzed population, the sensitization frequency to punein is lower than that observed for other allergens from the same source. In particular, among the subjects sensitized to at least one of the pomegranate pulp allergens, the frequency of IgE binding to punein was 5.6%, whereas higher values, corresponding to 59.9%, 19.3%, and 12.3%, were obtained for Pun g 1 (9k-LTP), the chitinase Pun g 14 (class III chitinase), and Pun g 7 (pommaclein), respectively.

Although the frequency of sensitization to the pomegranate PR-4 protein(s) is lower compared to that obtained for the other tested proteins, it has nevertheless been reported that it can sometimes cause severe symptoms. For instance, Buyuktiryaki and collaborators [5] reported the case of a boy from Turkey who experienced anaphylaxis following the ingestion of pomegranate. An investigation by immunoblotting assay, following electrophoretic separation, under non-reducing conditions, of a PPE, revealed the presence in his serum of IgE antibodies against two protein bands of 16 and 17 kDa. The authors of this study identified the IgE binding proteins as belonging to the PR-4 family, following a proteomic analysis, including mass spectrometry experiments. In fact, they obtained a peptide showing 100% identity with the corresponding region of PR-4 proteins from the genera *Prunus*, *Malus*, and *Chimonanthus*. In addition, they reported having obtained another peptide showing high identity with a region of the allergen Bra r 2 from *B. rapa*. Later, Hassan and Venkatesh [35] published a review describing allergens of the PR-4 family with apparent molecular masses of 28, 16, and 17 kDa on SDS-PAGE. Unfortunately, this review does not provide precise literature reference concerning these proteins. Considering that sometimes the apparent molecular masses on SDS-PAGE may be slightly distorted, the reported values (16–17 kDa) could identify the PR-4 IgE binding punein, which we observe to migrate at about 14 kDa.

### 2.7. Schematic Comparison of Punein and Prohevein

**Figure 6:**
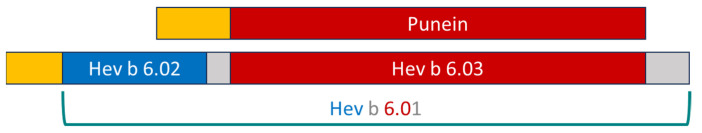
Schematic representation of punein with its signal peptide compared with prohevein (Hev b 6.01) linked to its signal peptide. Mature punein and hevein 6.03, containing the barwin module, are in red; signal peptides are in orange; hevein 6.02 is in blue; the fragments missed following the prohevein processing are in gray.

The comparison of punein amino acid sequence with that of the widely studied prohevein (Figure 6) by a simplified scheme may turn out to be of interest to make some points clear and collect further details on this pomegranate protein.

Literature reported [31] that pomegranate produces the preprohevein, which undergoes the removal of the signal peptide producing the prohevein (Hev b 6.01). Prohevein undergoes a post-translational processing, producing the N-terminal module known either as “hevein” or as “hev b 6.02”, and the C-terminal module (Hev b 6.03), plus some fragments that are lost. Several authors have reported that both hevein and the C-terminal domain of prohevein are allergenic and may be recognized by sera from latex-allergic individuals [32]. However, hevein is reported to be a lectin-bearing chitin-binding domain [32], described as the most important allergen impacting health care workers sensitized by rubber latex gloves [36]. As an important elicitor of latex-associated plant allergies, it is used as a recombinant fusion protein in diagnostic tests [37]. It is also worth noting that in the literature the term “hevein-like proteins”, is sometimes used to refer to the group of proteins containing the “hevein-like domain” (HLD). Therefore, in addition to prohevein, allergens such as Bra r 2, Hev b 11, and the kiwifruit Act c chitinase belong to this group. In contrast, the alignment of Figure 5 and the scheme in Figure 6 highlight that punein does not contain HLD.

## 3. Materials and Methods

### 3.1. Preparation of the PPE

Pomegranates (*Punica granatum*) used in this study were at the commercial ripening stage. The arils contained in each pomegranate fruit were manually separated into pulp and seeds. The pulp was homogenized in a blender (Tefal Optimo, Selongey, France) after the addition of 1 M NaCl (1:1 *v*/*w*) and stirred at 4 °C for 2 h. Next, the sample was centrifuged at 17,300× *g* for 45 min using a Sorval RC6 plus (Thermo Fisher Scientific, Osterode, Germany) and each supernatant, representing the PPE, was collected and stored at −80 °C until use.

The PPE was prepared at least 10 times from different fruit batches. Each PPE was analyzed by RP-HPLC (see below for details) because the protein profile was not constant, but the time of elution of proteins was always reproducible. Therefore, the PPE showing a high absorbance value of the peak eluted at 36 min were selected every time and used to purify punein.

### 3.2. Analysis by SDS-PAGE

The PPE and purified proteins were analyzed by reducing 15% SDS-PAGE [38] on a Bio-Rad Mini Protean apparatus (Segrate, Italy). The staining was carried out in 0.05% Coomassie R-250 brilliant blue in 40% methanol/10% acetic acid; the rinsing was performed in 40% methanol/10% acetic acid.

### 3.3. Determination of Protein Concentration

The protein concentration was determined by the BIO-RAD Protein Assay (Bio-Rad, Milan, Italy), using calibration curves made with bovine serum albumin [39].

### 3.4. Analysis by RP-HPLC

The PPE and some proteins were analyzed by RP-HPLC on a Vydac (Deerfield, IL, USA) C4 column (250 × 4.6 mm), using a Beckman System Gold apparatus (Fullerton, CA, USA). The elution was carried out by a multistep linear gradient of eluant B (0.08% TFA in acetonitrile) in eluant A (0.1% TFA) at a flow rate of 1 mL/min. The eluate was monitored at 220 and 280 nm. The separated fractions were manually collected and analyzed.

### 3.5. Amino Acid Sequencing

To identify the purified protein, automated N-terminal amino acid sequencing, using Edman degradation chemistry, was performed. In particular, the sample was loaded in a few μL on an automated Shimadzu protein sequencer PPSQ-33B (Shimadzu Corporation, Tokyo, Japan) and a cycle number was set at each experiment.

### 3.6. Obtainment of Protein Proteolytic Fragments

Internal fragments of native punein were subjected to sequencing. Some peptides were obtained by RP-HPLC separation of the protein subjected to proteolytic cleavages by bovine trypsin, following the instructions provided by the manufacturer (Roche Diagnostics, Mannheim, Germany). In particular, the digestion with trypsin was carried out at an enzyme:protein ratio of 1:20 (*w*/*w*) in Tris-HCl 0.1 M pH 8.5, at 37 °C for 3 h.

Additional peptides were obtained by digestion of the native protein with pepsin (Roche Diagnostics). This proteolysis was performed at an enzyme:protein ratio of 1:25 (*w*/*w*) in 5% formic acid, at 37 °C for 90 min. After each incubation, the obtained peptides were separated by RP-HPLC, manually collected, and some of them were analyzed by direct sequencing. Details about the RP-HPLC chromatography were the same as described above.

### 3.7. Mass Spectrometry Experiments

Mass spectrometric identification of the purified punein was carried out using the LC/MS Agilent single quadrupole system (Agilent 1260 Infinity II LC System, Santa Clara, CA, USA) equipped with a diode array detector combined with a dual electrospray ion source, a single quadrupole mass analyzer. The sample was analyzed on the column Aeris peptide XB-C18 (Phenomenex, Torrance, CA, USA) 100 mm × 4.6 mm, applying a gradient of eluent B (0.1% TFA in acetonitrile) in eluent A (0.1% TFA in water) from 5% to 70% in 30 min at a flow rate of 800 μL/min.

### 3.8. Bioinformatic Investigations

A primary structure similarity search in the UniProtKB, using the BLAST program, was performed on the server www.expasy.org. The databases used were UniProtKB Swiss-Prot, which contains high quality manually annotated and non-redundant protein sequences, and UniProtKB reference proteomes + Swiss-Prot, which also includes proteins derived from complete genome sequences. Details about protein molecules, including the molecular mass calculation on the basis of the primary structure, were obtained with the ProtParam tool (https://web.expasy.org/protparam/), on the Expasy platform. Predicted cleavage sites obtainable by enzymatic digestion of the purified protein were determined through PeptideCutter on the expasy.org server.

## 4. Conclusions

The presence of punein in pomegranate was observed some years ago through the analysis of a PPE, using an RP-HPLC separation followed by the direct sequencing of some proteolytic fragments of the protein. This protein was then registered in the UniProt database as punein. The IgE binding of this protein was mentioned in a previous publication [10], for comparison with other proteins, whereas the description of the purification of the punein and its structural characterization were postponed to a future paper. Therefore, it is described here.

A better structural and functional characterization of punein is possible only if a sufficient quantity of the protein is available to carry out the necessary experiments. Now, this paper reports for the first time the set up of a protocol that is useful to purify punein from its natural source in good amounts. Therefore, the availability of sufficient amounts of this protein will allow experiments of IgE binding in different populations and will make it possible to carry out in depth immunological characterizations, including allergenicity studies, which are not reported in this paper. At any rate, the data here described, although not conclusive on the structural and functional characteristics of punein, are useful in filling an information gap regarding pomegranate proteins belonging to the PR-4 family.

This study shows that punein belongs to class 2 of PR-4, since it contains the barwin domain and lacks the hevein one. The observation that some sequence regions of this molecule are at least partially conserved with respect to the C-terminal domain of prohevein, suggests that the IgE binding and allergenicity of punein deserve further investigation. The capacity to bind IgE, together with literature reports [5,10], make punein a protein whose effect on human health is worth studying. In fact, extensive and deep investigations could help the understanding of the clinical relevance of this protein at the worldwide level. In addition, the identification of punein expands the group of potential pomegranate allergens and may contribute to the improvement of diagnostic tests.

Some general remarks may also be made. The RP-HPLC chromatograms shown by Tuppo et al. [9] highlight a high variability of some pomegranate proteins, such as the allergens 9k-LTP Pun g 1 [8], the pommaclein Pun g 7 [9], the class III chitinase Pun g 14 [10], and the potential allergen Pun g 5 [10], corresponding to punein. This observation suggests that the frequency of IgE binding and allergenic reactivity to pomegranate may also be influenced by the variability in the expression of these proteins. In fact, some molecules, including some allergens, are not constitutively expressed proteins (non-CEP) [40], but they are rather proteins produced in response to offending factors, such as the pathogenesis-related (PR) proteins. These proteins can be allergenic and their amount in the source depends on the exposure to adverse factors, such as environmental and pathogenic attacks, which induce their synthesis (Factor-induced Expressed Proteins, FEP). Therefore, it is conceivable that the variability of the presence and concentration of some proteins, including the allergens Pun g 1, Pun g 7, Pun g 14, and potential allergens such as punein, are also influenced by differences in the exposure of pomegranate plants and fruits to pathogens, environmental stress, and climatic factors. This variability may at least partially explain the frequency of sensitization and allergic reactions to specific sources, such as pomegranate, within a population. Future studies could increase the knowledge of the influence of some factors on these mechanisms, that might be exploited to select, and possibly to produce, fruits with higher or lower amounts of specific proteins, depending on the objectives. In fact, the presence in a fruit of higher amount of a protein could be useful when the objective is the purification of that molecule, either for studies or for the use in diagnostics. In contrast, the presence of a low amount, or even the absence, of specific allergenic proteins could be useful to limit the sensitization towards some food allergens and to protect allergic patients with foods suitable for their diet.

## Figures and Tables

**Table 1:** Similarity searches in the UniProtKB database using the punein sequence as the query and setting the E-Treshold at 1000 and the taxonomy as Viridiplantae.

Query Sequence	Searched Database	Keyword (If Used to Make a Selection)	First on the List of Found Proteins	Accession Number
YHYYNPEENHWSLDDAK	UniProtKB Swiss-Prot		Punein (two fragments)	C0HKC6 (two fragments)
YHYYNPEENHWSLDDAK	UniProtKB reference proteomes + Swiss-Prot		Predicted PR-4 (entire punein)	A0A218X9Y8 (entire punein)
Punein (entire molecule)	UniProtKB Swiss-Prot	Allergen	Prohevein and Bra r 2	P02877 and P81729

**Table 2:** Amino acid sequence identity (%) between punein and the other PR-4 mature proteins shown in the alignment of Figure 5. The identity values were calculated using the Clustal Omega algorithm on the UniProt website by comparing the amino acid sequences two by two.

	Punein	Wheatwin-2	Wheatwin-1	Barwin	Bra r 2	Prohevein
Punein	100					
Wheatwin-2	55.00	100				
Wheatwin-1	54.17	98.40	100			
Barwin	54.17	96.80	95.20	100		
Bra r 2	44.83	75.86	74.14	79.31	100	
Prohevein	55.00	68.85	69.67	66.39	67.03	100

## Data Availability

The original contributions presented in this study are included in the article. Further inquiries can be directed to the corresponding author.
